# Central Post-Stroke Pain: An Integrative Review of Somatotopic Damage, Clinical Symptoms, and Neurophysiological Measures

**DOI:** 10.3389/fneur.2021.678198

**Published:** 2021-08-18

**Authors:** Daniel Fernando Arias Betancur, Maria da Graça Lopes Tarragó, Iraci Lucena da Silva Torres, Felipe Fregni, Wolnei Caumo

**Affiliations:** ^1^Graduate Program in Medical Sciences, School of Medicine, Federal University of Rio Grande do Sul (UFRGS), Porto Alegre, Brazil; ^2^Laboratory of Pain & Neuromodulation, Clinical Research Center, Hospital de Clínicas de Porto Alegre (HCPA), Porto Alegre, Brazil; ^3^Physical Medicine and Rehabilitation Service, Hospital de Clínicas de Porto Alegre (HCPA), Porto Alegre, Brazil; ^4^Pharmacology of Pain and Neuromodulation: Pre-clinical Investigations Research Group, Federal University of Rio Grande Do Sul (UFRGS), Porto Alegre, Brazil; ^5^Laboratory of Neuromodulation and Center for Clinical Research Learning, Physics, and Rehabilitation Department, Spaulding Rehabilitation Hospital, Boston, MA, United States; ^6^Pain and Palliative Care Service, Hospital de Clínicas de Porto Alegre (HCPA), Porto Alegre, Brazil; ^7^Department of Surgery, School of Medicine, Federal University of Rio Grande Do Sul (UFRGS), Porto Alegre, Brazil

**Keywords:** stroke, neuropathic pain, TMS, cortical excitability, cerebellum

## Abstract

**Introduction:** The physiopathology of central post-stroke pain (CPSP) is poorly understood, which may contribute to the limitations of diagnostic and therapeutic advancements. Thus, the current systematic review was conducted to examine, from an integrated perspective, the cortical neurophysiological changes observed *via* transcranial magnetic stimulation (TMS), focusing on the structural damage, and clinical symptoms in patients with CPSP.

**Methods:** The literature review included the databases EMBASE, PubMed, and ScienceDirect using the following search terms by MeSH or Entree descriptors: [(“Cerebral Stroke”) AND (“Pain” OR “Transcranial Magnetic Stimulation”) AND (“Transcranial Magnetic Stimulation”)] (through September 29, 2020). A total of 297 articles related to CPSP were identified. Of these, only four quantitatively recorded cortical measurements.

**Results:** We found four studies with different methodologies and results of the TMS measures. According to the National Institutes of Health (NIH) guidelines, two studies had low methodological quality and the other two studies had satisfactory methodological quality. The four studies compared the motor threshold (MT) of the stroke-affected hemisphere with the unaffected hemisphere or with healthy controls. Two studies assessed other cortical excitability measures, such as cortical silent period (CSP), short-interval intracortical inhibition (SICI), and intracortical facilitation (ICF). The main limitations in the interpretation of the results were the heterogeneity in parameter measurements, unknown cortical excitability measures as potential prognostic markers, the lack of a control group without pain, and the absence of consistent and validated diagnosis criteria.

**Conclusion:** Despite the limited number of studies that prevented us from conducting a meta-analysis, the dataset of this systematic review provides evidence to improve the understanding of CPSP physiopathology. Additionally, these studies support the construction of a framework for diagnosis and will help improve the methodological quality of future research in somatosensory sequelae following stroke. Furthermore, they offer a way to integrate dysfunctional neuroplasticity markers that are indirectly assessed by neurophysiological measures with their correlated clinical symptoms.

## Introduction

Stroke is the second leading cause of death in the world (1, 2). Among those who survive, motor and somatosensory sequelae compromise the functional capacity and quality of life in many individuals (3–5). In general, one can define two types of pain after stroke: pain associated with peripheral mechanisms (e.g., musculoskeletal, spastic pain, headache, and shoulder pain) and neuropathic central post-stroke pain (CPSP) (6). Although, CPSP is one of the primary sequelae following stroke, there is a gap in understanding its pathophysiology and a diagnostic definition (7). Dejerine and Roussy (8) performed the first description of CPSP in 1906. They described the clinical–anatomical correlation of patients diagnosed with a thalamic stroke who presented a syndrome characterized by intense pain, changes in superficial and deep sensorial perception, mild hemiplegia, choreoathetoid movements, astereognosis, and hemiataxia. Initial studies have linked CPSP to stroke in the thalamus, specifically the pulvinar, the ventral posteromedial, and posterolateral nuclei (9, 10). However, later studies described CPSP in lesions located in the lateral medulla (11), lenticulo-capsular area (12), pons, and in cortical areas (insula and operculum) (13, 14).

Although, there has been enormous progress in stroke treatment in recent decades, the rehabilitation of those who survive a stroke remains a challenge, specifically the recovery of disability, and well-being. Among these sequelae is CPSP, which has been shown to be slightly improved by pharmacological treatments. There is limited literature concerning this neuropathic pain category, primarily focusing on diagnosis and treatment. Thus, a better understanding of the neuroplasticity process might help progress in this field. In this regard, there is an urgent need to conduct studies investigating markers with diagnostic and prognostic potential to assist in the treatment of CPSP. This systematic review aimed to gather data from the literature concerning the physiopathology of CPSP and to critically examine these data to assist in planning future studies that may be able to help in the neurorehabilitation and optimization of functional recovery in individuals affected by CPSP. Specifically, the current review explored, from an integrative perspective, the relationships of the anatomical areas, clinical symptoms, and the cortical excitability (CE) parameters indexed by transcranial magnetic stimulation (TMS) measures. The TMS measures included motor threshold (MT), motor evoked potential (MEP), short intracortical inhibition (SICI), intracortical facilitation (ICF), and cortical silent period (CSP). From this dataset, we hope to offer additional information to advance this field of knowledge and open a new avenue for the treatment and rehabilitation of individuals affected by CPSP.

## Principal Concepts in CPSP

### Prevalence and Incidence

It is estimated that the prevalence of CPSP ranges from 1 to 35% (15). This broad estimate is possibly due to variabilities in the definition of this pain category, the inclusion criteria, and the length of patients' evaluation post-stroke (16). For example, the prevalence of CPSP was 25% in an earlier study that included 63 patients with vascular damage to the medulla's lateral part (11). In another study, the prevalence of CPSP was 1% in patients whose assessment was 16 months post-stroke (17). Regarding the incidence of CPSP, a study found that, in an initial sample size of 207 stroke patients, 8% developed symptoms compatible with central neuropathic pain during the first year after stroke (18). However, commencement of symptoms showed extensive variations among studies. Nasreddine and Saver (19) found that most patients began experiencing symptoms within the first 6 months. In contrast, another study found cases in which CPSP appeared up to 10 years after the event (20). According to these data, it is possible that the course of CPSP across time can be variable, with several reports of some patients experiencing symptoms for years or throughout their entire life (6).

### Signs and Clinical Symptoms

CPSP can be continuous or intermittent, with pain described as burning, throbbing, pressure, or freezing (16, 21). According to Boivie's review (22), abnormalities in stimulus perception were defined as hyperesthesia, hyperalgesia, hypoesthesia, paresthesia, dysesthesia, allodynia, or hyperpathy and may be associated with temporal and spatial summation. Bashir et al. (23) diagnosed CPSP in six patients, with a primary pain description of a burning sensation in 62.5% of them, followed by sensation of electric shock in 25%. Additionally, this study found as aggravating factors movement of the extremity, contact with heat/cold, and psychological stress. The predominant sensory abnormalities identified were tactile allodynia and hyperalgesia, both with a prevalence of 35.7%. Other authors have also emphasized impairment in the perception of stimuli by needle prick, temperature, or touch, and the lower frequency in vibration perception and joint positioning (16). Typically, pain is restricted to the anatomical area with somatosensory abnormalities (14) and impairment of body segments, both proximal and distal, which are correlated with the anatomical location of the lesion within the central nervous system (CNS) (20, 24). In capsule-lenticular lesions, there is a higher prevalence of pain in the lower limbs than in the face or upper limbs (12). In thalamic lesions, contralateral hemi-body manifestations are the most frequent (25). Conversely, in medullary injuries, the signs and symptoms depend on the location of damage, with distinct symptoms if the lesion is lateral or medial. In lateral lesions, facial impairment symptoms might be either ipsilateral or contralateral, associated with pain descriptors of either burning or cold. In medial lesions, the most commonly reported impairment is in the trunk and extremities, with descriptors such as numbness and/or tingling (26).

### An Integrative View on the Diagnosis of CPSP

CPSP is considered a chronic pain condition that has mobilized clinicians and researchers to improve the diagnostic criteria. Thus, the International Association for the Study of Pain (IASP) Committee established a panel of experts with a particular interest in neuropathic pain (NeuPSIG) to review a neuropathic pain classification system created in 2008 (27). According to this committee's guidelines, neuropathic pain diagnosis should include the patient's clinical history of neurological damage, which must have somatotopy, signs, and symptoms with correspondent plausibility to the damage in the CNS. Additionally, confirmatory tests are needed to identify such damage in the somatosensory system (27).

As specified in the IASP Committee's criteria, CPSP is manifested contralateral to the affected hemisphere. If the lesion is located in the brainstem, the pain distribution may occur on the ipsilateral side of the face (27). The diagnosis should be guided by history and clinical evaluation, complemented with neuroimaging data to increase diagnostic accuracy (28). It has been demonstrated that sensory evaluation can serve as a predictor of the development of CPSP. Post-stroke dysesthesia, allodynia, or hyperalgesia is associated with a 4.6-fold increase in the probability of CPSP development in the first 6 months following a brain vascular event. Similarly, the presence of early pain, or dysesthesia, and a reduced or an absent sensation to needle stick or cold increase the probability of post-stroke neuropathic pain 8-fold (29).

Despite clinicians' and researchers' best efforts to systematize the diagnostic criteria, this category of neuropathic pain after stroke remains a challenge, primarily due to the heterogeneity of the clinical symptoms. Both Klit et al. (28) and Hansen et al. (30) have proposed diagnostic criteria used to assess patients with CPSP. These criteria are presented in Table 1.

**Table 1:** Diagnostic criteria for CPSP.

**Klit et al. (28)**	**Hansen et al. (30)**
1) Pain in an area of the body with somatotopic correspondence to the CNS lesion. 2) Suggestive history of stroke and the appearance of pain can happen early or with some delay over time. 3) Confirmation of CNS lesion by image or positive or negative sensory signs, with somatotopic correspondence to the area of the lesion. 4) Exclusion of other causes that may explain the painful symptoms. The supportive criteria include pain with no direct relation to movement, inflammation, or tissue damage; descriptors of neuropathic pain like burning, electrical shock, painful cold, aching, pressure, sting, pinprick, and needle; and complaint of allodynia or dysesthesia to the touch or cold.	1) Development of pain with onset of or after the stroke. 2) Pain located on the stroke-affected side of the body. 3) No other plausible cause of the pain, including pain isolated to the shoulder joint and nearby region.

### Physiopathology Theories and Plasticity Role in CPSP

The pain pathway comprises a complex network of axonal projections to different brain regions. Such connections include the ventral and dorsal medullary reticular formation, the dorsal spine nucleus, the parabrachial area, the locus coeruleus, and the periaqueductal gray matter. Additionally, the lateral and medial thalamus, anterior pretectal nucleus, amygdala, and the hypothalamus are also part of the neural network of pain processing. The ascending path is formed by two parallel pain pathways, divided into the medial and lateral systems. The medial system comprises the spinohypothalamic, spinoamygdalar, medial spinothalamic, and spinoreticular tracts. It has connections with the limbic, prefrontal, and cingulate cortices. Furthermore, it is responsible for transmitting information associated with the affective, motivational, and autonomic responses to pain. In contrast, the lateral pathway comprises the spinothalamic tract. This tract transmits information to the lateral thalamus and, later, to the primary and secondary somatosensory cortices, which are responsible for identifying the nociceptive stimulus quality, pain location, and intensity (31). Although, the neuroanatomical connections are known, the mechanisms responsible for the emergence of CPSP remain unclear; however, it is known that they transcend the effect of structural damage. According to theory, a central mechanism is a functional imbalance between the excitatory and inhibitory systems in pain pathways, which can be related to specific neural circuits associated with the neuroanatomical lesion areas.

In 1911, Henry Head and Gordon Holmes (32) proposed the disinhibition theory, intending to explain the change in the perception of painful and non-painful stimuli in individuals with injury to the lateral thalamus. According to these authors, a lateral nucleus injury would lead to loss of cortical control mechanisms. Thus, thalamus hyperactivity and the consequent exacerbated response to stimuli would occur. Later, Craig et al. (33), based on this theory, proposed an idea of imbalance between the output of the thermosensory area in the insula and the limbic network associated with thermoregulatory motivation as a consequence of CNS lesion. According to this theory, lesions of the lateral lamina I spinothalamocortical pathway, connected to the parieto-insular cortex by posterolateral thalamus projections, would be related to the disinhibition of nociceptive polymodal activity. This disinhibition takes place in the medial lamina I spinothalamocortical pathway, which is connected to the anterior cingulate cortex. Therefore, there would be loss of thermo-sensorial integration, which manifests itself as a burning sensation and produces exacerbated responses to temperatures previously perceived as harmless.

Other studies corroborate the idea that damage to the spinothalamic pathway is a central mechanism of CPSP. In 1989, Boivie et al. (34) found that injury at any level in this pathway could be responsible for the emergence of CPSP. Vartiainen et al. (35) also demonstrated that impairment of the spinothalamocortical pathway is a predictive factor independent of the development of central pain. In addition to structural lesions of this pathway, CPSP may also occur due to brain plasticity changes (36). This theory posits that dysfunctional neuroplasticity is a central mechanism of CPSP and finds support in the pathophysiology of other neuropathic pain conditions, in which the pathological phenomenon of spontaneous pain is linked to maladaptive mechanisms of cortical and thalamic hyperexcitability (14). Gritsch et al. (37), in a mouse study, showed that mechanical hypersensitivity in CPSP does not depend on the expressions of type I vanilloid-type receptors (TRPV1) on nerve fibers and neurokinin-1 receptors of the spinal cord. They suggest that central pain is due to lateral thalamus hyperexcitability, which is associated with the expressions of calcium-voltage-dependent channels and changes in the GABAergic inhibitory system. Similar results in other animal studies have shown a possible association in the development of CPSP, with increased connections in the affected hemisphere, between the mediodorsal nucleus of the thalamus and the amygdala (38). In addition, in a study by Kuan et al. (39), aberrant neuronal activity in the pathway between the medial thalamus and cingulate cortex, having the brain-derived neurotrophic factor (BDNF) as a mediator, was observed. The authors suggest that, in this type of thalamocortical dysrhythmia, an imbalance in the activity between the GABAergic and glutamatergic systems is involved.

**Figure 1:**
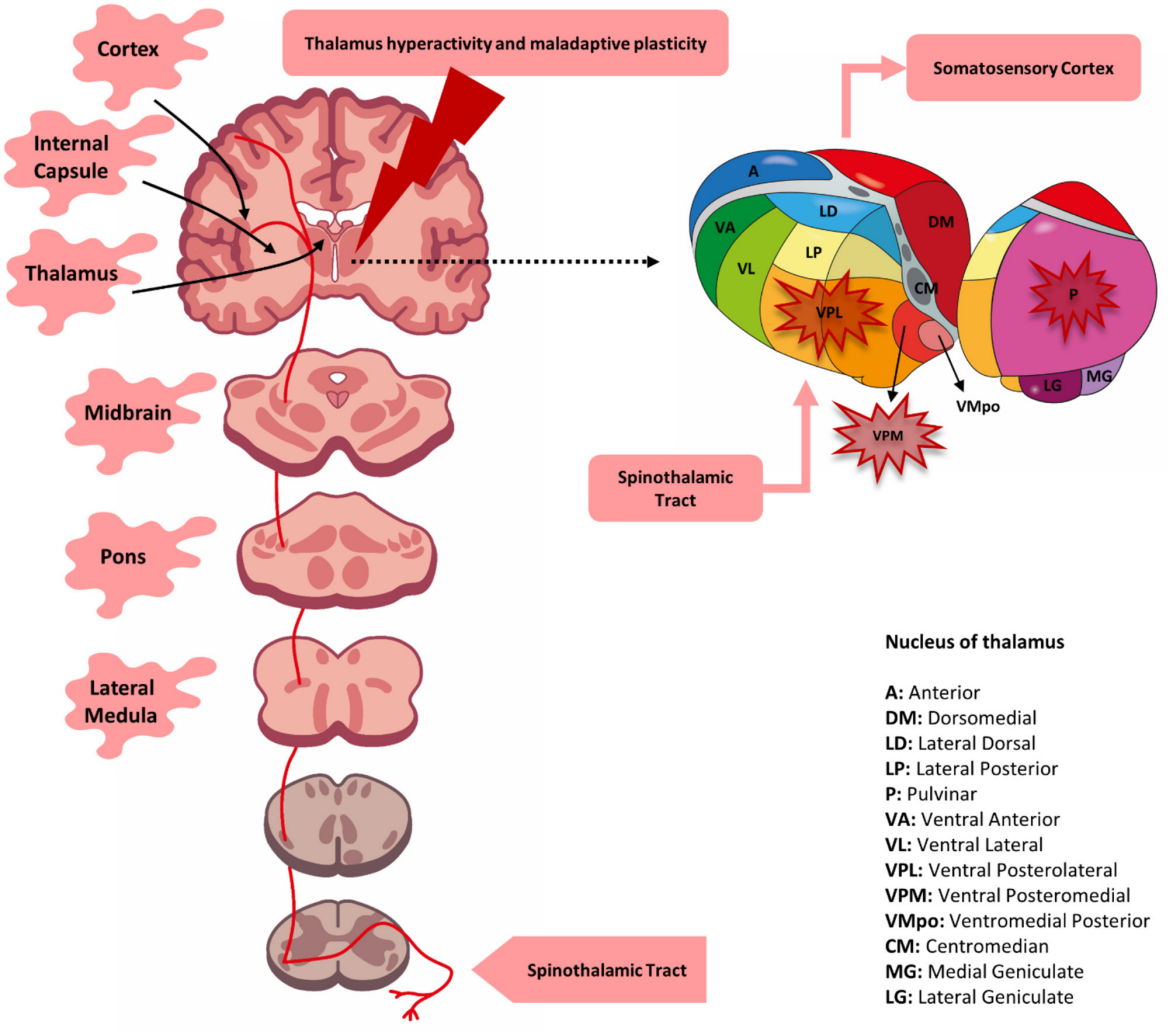
Lesion site associated with central post-stroke pain (CPSP). A lesion located at any level of the spinothalamic tract (*red line*) may be responsible for the development of post-stroke pain. Although, involvement of the thalamus (primarily the ventral posteromedial nucleus, posterolateral nucleus, and possibly the pulvinar nucleus) was initially determined to be solely responsible for pain, it was later determined that lesions of the lateral medulla, pons, lenticulo-capsular, and cortex could also be associated. CPSP is possibly the result of the loss of somatosensory integration along with changes in functional and cortical plasticity.

To corroborate this idea, studies using magnetic resonance imaging have shown that individuals with CPSP present a characteristic pattern of cortical atrophy in different regions, which include the temporal, secondary somatosensory, insular, and ventrolateral prefrontal cortices, as well as the nucleus accumbens. These changes indicate anatomical variations that can explain maladaptive alterations associated with the affective component of pain and with discriminatory sensory impairment (36). A later study, using diffusion tensor imaging, found white matter microstructural changes in areas associated with pain processing (i.e., the anterior cingulate cortex, posterior insula, thalamus, and the somatosensory cortex). Also, this study found increased functional connectivity in the anterior cingulate cortex and decreased connectivity in the somatosensory cortex (40). Thus, an integrative approach using neurophysiological measures to understand the relationship between the area of neuroanatomical damage and the physiological state could help diagnose and direct new therapeutic models (37). Figure 1 illustrates the lesion sites involved in the physiopathology of CPSP.

Despite advances in psychopharmacological treatments over the last few decades, their efficacy is limited, and there is a high incidence of adverse effects in CPSP (41, 42). Accordingly, it is possible to theorize that this low success rate is related to the limited power of pharmaceuticals to modify the maladaptive neuroplasticity in pain processing pathways. Thus, the motivation to understand dysfunctional neurophysiological processes grows to guide the search for complementary non-pharmacological options. The above-mentioned background gives support to explore how non-pharmacological approaches, such as non-invasive or invasive brain stimulation (43), or other therapeutic approaches, such as hypnotherapy, mindfulness, or cognitive–behavioral therapy, can change pain sensitivity. In a pragmatic view, advancement in the therapeutic field may be more likely to occur if we improve the capacity to characterize the phenotypes of CPSP and evaluate the dysfunction in neuroplasticity processes by neurophysiological measures. Among the tools available, TMS and functional neuroimaging permit us to assess the impacts of treatments on dysfunctional neuroplasticity processes that lead to and maintain CPSP.

## TMS Measurements as a Potential Biomarker

TMS is a non-invasive, versatile, and painless tool for measuring different cortical parameters *in vivo* (44). TMS measures provide indirect information about the systems responsible for modulating interneuron activity, such as the GABAergic, glutamatergic, and cholinergic pathways. However, these systems are affected by pathological processes in the CNS (45, 46).

Among the TMS measures, MEP is the main parameter used in clinical neurophysiology. It represents the excitability/conductivity and the integrity of corticospinal pathways (47). It results from a unique TMS pulse, delivered through a coil located over the scalp, to generate an eddy current that activates transsynaptic pyramidal neurons and results in a muscular action potential recorded through surface electrodes over selected target muscles (48). Another important measure in the scientific field, as well as in clinical practice, is cortical MT. This measure permits the calculation of the individual intensity of the TMS pulse in the subsequent protocol (e.g., an intensity of 80–120% of the MT) (49). This measure is assessed when the target muscle is at rest. The resting MT (RMT) is the minimal intensity of the TMS pulse to produce an MEP of at least 50 mV in 50% of the attempts (47, 49). Alternatively, active MT (AMT) is assessed during a slight muscular contraction (~20%) of the target muscle (47, 49). It is defined as the minimal pulse intensity to elicit an MEP ≥ 200 μV in 50% of the attempts. It corresponds nearly to the threshold necessary to induce activation in fast-conducting neurons (46, 49). While the RMT infers glutamatergic synaptic connections, the AMT is voltage-gated cation channel-dependent (50, 51) and infers axonal excitability (52).

TMS measures can also be helpful in the evaluation of inhibitory physiological phenomena. After applying a TMS pulse with suprathreshold intensity over the motor cortex while the subject performs a sustained muscular contraction on the contralateral side of the body, a period of electrical silence on the electromyography activity occurs following the MEP (49). This parameter is referred to as a CSP, and it is mediated, in its early and late phases of EMG suppression, by spinal and supraspinal inhibitory mechanisms, respectively (53).

In addition to a single TMS pulse, a paired-pulse TMS can be useful in assessing inhibitory and facilitatory intracortical circuits. This paradigm comprises a subthreshold conditioning stimulus (CS) followed by a suprathreshold test stimulus (TS). The interstimulus intervals (ISIs) vary from 1 to 20 ms. Furthermore, the MEP amplitude produced by the paired pulse is compared with the TS alone that works as a reference condition (45, 49). In practice, the paired pulse applied with an ISI of 1–6 ms and the corresponding MEP amplitude result from a cortical inhibitory phenomenon known as SICI (47). The SICI has two phases—the short ISI (around 1 ms) is associated with neural refraction, while the second phase of 2.5 ms is the long interval (54, 55)—and reflects synaptic inhibition mediated by GABA type A receptors (56). However, if an ISI of 7–20 ms is applied, the resulting increase in the MEP amplitude is denominated as ICF (57, 58). The facilitation phenomenon is related to excitatory glutamatergic circuits. Both SICI and ICF reflect the activity of intracortical circuits dependent on the GABA/glutamate balance (47).

MEP inhibition as a consequence of electrical peripheral nerve stimulation (usually the median nerve at the wrist or with a digit), preceding a subsequent suprathreshold TMS in the contralateral motor cortex, with ISI ranging between 20 and 25 ms, is known as short-latency afferent inhibition (SAI) (48). Pharmacological studies have shown that SAI is related to cholinergic (59) and GABAergic circuits (60). This measure can assess the inhibitory circuits between hemispheres (interhemispheric inhibition) and cerebellar–cortical connections. Interhemispheric modulation can be tested by applying two TMS pulses at ISIs in each hemisphere (61). In the same way, cerebellar brain inhibition (CBI) is assessed when the MEP amplitude is reduced after a TMS pulse in the cerebellum, followed by a second pulse applied in the motor cortex (M1) (62).

Different studies have shown that TMS measurements can be helpful to understand the development, prognosis, and therapeutic approach of various pathological processes and specific diseases. For instance, in the case of vascular dementia (VaD) and Alzheimer's disease, a decrease in SAI has been observed (25% of subjects in VaD). Additionally, pathological reduction of the MT and intracortical inhibition (ICI) are parameters that indicate imbalance in the glutamatergic, GABAergic, or the cholinergic system (63).

Likewise, in patients with pain, cortical excitability measurements can reflect descending pain modulatory system function, and their variations are associated with the clinical response to exogenous approaches, such as physical activity, TMS, or transcranial direct current stimulation (64, 65). Post-stroke TMS neurophysiological measures present a prognostic value of adaptive mechanisms in both acute and chronic phases (66). Thus, variations in the CE measures as a reflection of maladaptive plasticity may be fundamental in building a critical integrative perspective of CPSP, either in diagnosis or treatment.

### TMS Measurements in Stroke

In healthy subjects, a study by Mills and Nithi (67) evaluated MT and its relationship with several variables, such as gender, age, and the hemisphere assessed. They demonstrated a positive correlation in the MT value and no differences in the means between hemispheres. Conversely, after stroke, the MT is in the normal range in the unaffected hemisphere, both in the acute (68–70) and chronic phases (69, 71). However, in the affected hemisphere, the MT is usually increased compared to the unaffected hemisphere or compared to healthy subjects, either in the acute (68–70, 72, 73) or subacute phase (70, 74, 75). After the subacute phase, the MT decreases progressively over time (76). This phenomenon is associated with a cortical recovery process from axonal remyelination secondary to oligodendrocytic cellular precursor maturation (77). Furthermore, some studies have confirmed the presence of asymmetries in MT according to the location of the lesion, whether in cortical or subcortical structures. A study by Delvaux et al. (78) evaluated MT in cortical and cortico-subcortical stroke and did not identify a statistically significant difference in the comparison of the affected hemisphere with controls. Alternatively, other studies have found increased MTs in subcortical lesions (74, 79). The purported biological cause of this finding was damage to the neural fibers essential for responsiveness to TMS stimuli (80).

Like MT, MEP presents changes when evaluated in the affected hemisphere of stroke patients. In general, MEP has a decreased amplitude when compared to the amplitude of the unaffected side or the same hemisphere of healthy subjects (70, 73, 81). However, in some cases, a progressive MEP increase related to motor recovery of the participant was identified (69, 82). Usually, in the unaffected hemisphere, changes in MEP have not been observed (69, 70). Despite this, several studies have shown an increase in the acute phase after the stroke, with a subsequent tendency to normalize over time (78, 83). These initial cortical changes, located contralateral to the lesion, may occur due to a decreased GABAergic inhibitory activity and to alteration in transcallosal balance, both of which are consequent to neuronal ischemia (78).

MEP can be fundamental in understanding and directing the rehabilitation of patients after stroke. This is a neurophysiological measure that provides information related to corticospinal tract function and is capable of predicting the motor recovery of patients in the first weeks after injury (70, 76, 84). Thus, besides having prognostic properties, the absence or presence of MEP during the evaluation of a specific muscle may suggest the beginning of a rehabilitation plan to recover muscle functionality in patients with integrity, or at least some level of corticospinal pathway function (presence of MEP), or the implementation of compensatory treatment in cases of insufficient response (reduced or absent MEP) (85). Additionally, there is the possibility that MEP responses following the initial physical therapy sessions (goal-oriented task) can provide information on the therapeutic effectiveness of the intervention in the acute phase (86).

Like the previously mentioned cortical measurements of stroke, SICI, ICF, and CSP present specific patterns. Following stroke, SICI is reduced (87). This finding seems to be related to the structural damage severity and vascular lesion location. Huynh et al. (88) explored the relationship between the severity of brain injury and the SICI level in the affected hemisphere. They showed that the reduction in SICI was proportional to the degree of impairment due to sequelae after the stroke and found decreased SICIs in both cortical and subcortical lesions, leading to a more significant reduction in those with cortical lesions than in controls. Conversely, studies that have investigated the SICI in the unaffected hemisphere showed a decrease in this measure associated with the cortical location and the initial phase of stroke. However, the measure was not related to lesion extension or patients' motor recovery (89). A plausible explanation for this phenomenon was cortical plasticity reorganization as a response to vascular injury (90, 91) and loss of interhemispheric relationship integrity as a consequence of the imbalance of facilitation and inhibition interactions between the hemispheres (92). In the chronic phase of stroke, although, a decrease in SICI has been shown (93, 94), a meta-analysis conducted by McDonnell and Stinear (95) found an inconsistent SICI pattern when the affected hemisphere was compared with the unaffected hemisphere and healthy controls. Concerning ICF, the impact of acute and chronic stroke on this measure is less clear. There were no differences or consistency between the hemispheres or in the comparison with healthy controls at any time (95–97).

Despite CSP presenting significant inter-individual variability, small intra-individual differences between hemispheres were found. Therefore, its measurement allows an adequate assessment of unilateral changes (98). Regarding CSP behavior in stroke, studies have found that CSP tends to be prolonged in the acute phase after stroke (1–7 days) (87, 95, 99, 100). This finding could be interpreted as a consequence of the reduced ICI of inhibitory interneurons. It also reflects the presence of adaptive processes that favor an intracortical excitation state (101). Conversely, in subacute and chronic stroke, various CSP patterns (e.g., regular, shortened, or prolonged) were found. The authors suggest that this variability is due to the influence of variables such as lesion location or the presence of spasticity (98). In focal lesions of the motor cortex, the CSP may be decreased or absent due to impairments in cortical inhibitory interneurons (102). However, cortical and subcortical lesions distant from this region (motor cortex) extend the CSP due to cortical disinhibition related to the loss of the modulating projections to the motor cortex (99, 103). The presence of spasticity in patients in the chronic phase of stroke was related to a shortening of the CSP due to the decreased activity of the inhibitory circuits (104, 105).

### TMS Measurements in Pain

In cases of pain not explicitly related to stroke, we can better characterize the pathophysiological mechanisms involved in its development and evolution with the help of TMS measurements in the M1. When comparing acute and chronic pain of different etiologies, we found a critical variability in the MEP and MT. In acute experimental pain, the use of varying nociceptive stimuli, such as heat (106), cold (107), and capsaicin (108), reduced the MEP. This result was confirmed by data from a meta-analysis conducted by Burns et al. (109). The meta-analysis evaluated the effect and changes in cortical activity in the brain regions corresponding to the primary somatosensory cortex (S1) and primary motor cortex (M1) in acute myofascial pain. They found that the MEP decreased during the application of a painful stimulus (hypertonic saline solution and ascorbic acid). According to the authors, the cortical inhibition phenomenon in the M1 region occurred to limit motor activity and to avoid worsening the tissue injury and increasing pain.

In chronic pain, a reversal of this phenomenon may occur with increased MEP. This amplified MEP response in chronic pain has been interpreted as a cortical reorganization due to an imbalance between the inhibitory and excitatory systems (110–112). Interestingly, although, the MT increased in some cases (113, 114), it tended to remain unchanged in both acute (115) and chronic pain (65, 116) in most studies.

In addition to the MEP and MT, there are variations in other intracortical measurements associated with the pain that emerges during different pathological processes. Salo et al. (107) used an experimental model of acute pain in which a stimulus with cold water was applied to the right hand to simulate the effect of neuropathic pain on cortical activity. The study showed an increase of SICI in TMS evaluation. Another study on acute myofascial pain found an association with decreasing ICF (117). Moreover, a review and meta-analysis conducted by Parker et al. (118) on subjects with chronic pain demonstrated a significant reduction in CSP duration and SICI amplitude and an increase in ICF. Similarly, results were found that point to decreases in the CSP and ICI in different pathologies. Among them were osteoarthritis, myofascial pain, fibromyalgia (112), rheumatoid arthritis (114), CPSP, syringomyelia, brachial plexus injury, and median nerve peripheral neuropathy (65).

Measures of the CE parameters have already been shown to be important in the study and understanding of endogenous analgesic systems in healthy individuals and in patients with chronic pain. According to Granovsky et al. (64), in healthy individuals, there is a positive correlation between the amplitude and duration of MEP and the antinociceptive mechanisms evaluated by conditioned pain modulation (CPM). However, in chronic pain, MEPs of greater amplitude recorded in individuals with myofascial pain syndrome had no relation with a more efficient CPM response (111). Lefaucheur et al. (65) highlighted the importance of understanding the interaction between antinociception phenomena and CE. The therapeutic use of high-frequency TMS (10 Hz) in chronic neuropathic pain produced adequate analgesic responses related to ICI increase. Changes in ICI after repetitive TMS were linked to the recovery of GABAergic neurotransmitter activity (119).

### Cerebellum and TMS Measurements

**Figure 2:**
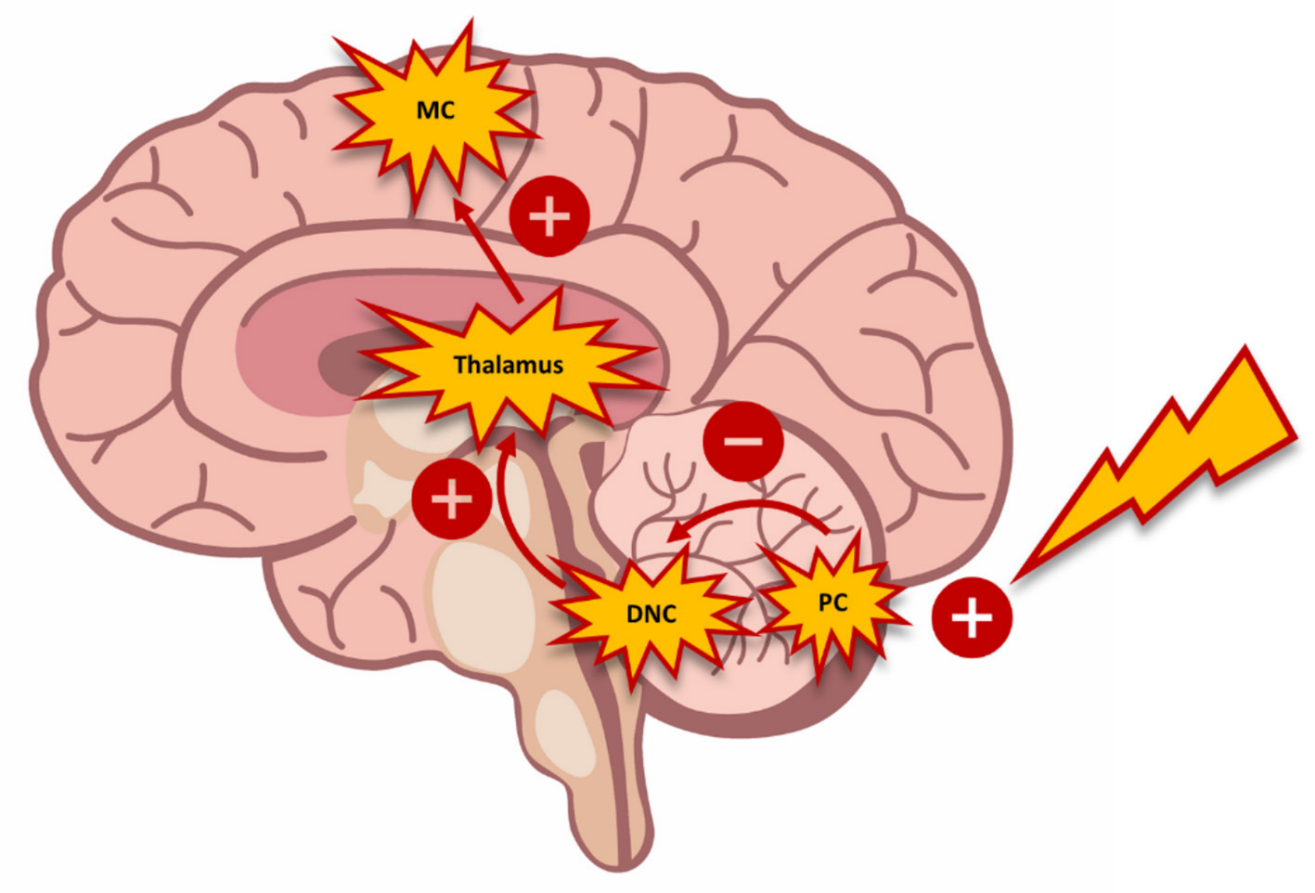
Schematic diagram demonstrating the interaction between the cerebellum and the motor cortex. The activation of Purkinje cells (*PC*) with inhibitory connections suppresses exiting stimuli from the deep cerebellar nuclei (*DCN*) and the ventrolateral nucleus of the thalamus toward the motor cortex (*MC*). The inhibition of this circuit triggers changes in motor control, which can be measured through transcranial magnetic stimulation.

The TMS model of paired stimulus permits quantification of the inhibitory tonus that the cerebellum exerts on the motor cortex, which is referred to as cerebellar brain inhibition (CBI) (62). Daskalakis et al. (120) found an inversely proportional relationship between SICI and CBI in healthy subjects. The interaction between the cerebellar and cortical circuits was explained by the activation of Purkinje cells with inhibitory activity by the magnetic impulse (121). Thereby, this inhibitory activity suppresses the excitatory stimuli from deep cerebellar nuclei and the ventrolateral thalamus nucleus. This chain effect could explain the decrease observed in the expression of cortical inhibitory mechanisms (i.e., SICI) (Figure 2). Conversely, in this same study, the researchers observed an increase in ICF dependent on the decrease in SICI. The authors interpreted that these findings were due to the imbalance between the excitatory and inhibitory cortical circuits (120).

In patients with CPSP, the effect of the cerebellum on cortical activity in the M1 may be relevant if we consider that, by adaptive mechanisms, pain induces motor changes that seek to protect the body and avoid nociceptive stimulus (122). Although, previous studies have focused on assigning this response to cortical areas (123), recent studies have linked this response to the cerebellum (specifically the posterior lobules VIe and VIIb). These cerebellar areas are associated with the anterior cingulate cortex, supplementary motor area, and the thalamus. Therefore, it is plausible that this adaptive motor response is due to the interplay of the regions involved in pain processing and the cerebellar circuits (124).

## Methods

The methodology of the current systematic review was based on the PRISMA (Preferred Reporting Items for Systematic Reviews and Meta-Analysis) guidelines. There has not been a previously published protocol in PROSPERO (International Prospective Register of Systematic Reviews) before the development of this study.

Relevant studies were sought in EMBASE (from 1993), PubMed (from 1996), and ScienceDirect (from 1997) databases. The MeSH or Entree terms used, and their combinations, were as follows: [(“Cerebral Stroke”) AND (“Pain” OR “Transcranial Magnetic Stimulation”) AND (“Transcranial Magnetic Stimulation”)] (through September 29, 2020).

### Criteria for Inclusion and Exclusion and Quality Analysis of Studies

We defined the following criteria to include a paper to the meta-analysis: studies in humans; written in English, Spanish, or Portuguese; the focus was on CPSP; use of TMS measures to evaluate cortical function; and quantitative results were provided. No filter was applied concerning the publication year or the study design.

Two authors independently evaluated and selected the included studies. If there was disagreement, those specific cases were discussed with a third evaluator. After the initial identification of the studies using the search strategy, duplicate articles were excluded and a second screening was performed, in which reports on animals and studies that were not related to the research question were discarded. Studies that included the phrases “post stroke pain,” “poststroke pain,” “post-stroke pain,” “central pain,” “central neuropathic pain,” “Dejerine–Roussy syndrome,” “thalamic pain,” “thalamic syndrome,” “Wallenberg syndrome,” and “central and peripheral neuropathic pain” were considered relevant. In the next step, the abstracts of 297 articles were evaluated to identify and exclude studies that did not use TMS as a therapeutic or diagnostic measure. The texts were then thoroughly evaluated; if the study was eligible, the following information was extracted: study category, sample size, mean age, stroke location, degree and duration of pain, TMS protocol, and the evaluated TMS measures.

### Bias Risk Assessment

Two authors independently evaluated the risk of bias related to the methodological aspects of each study according to the guidelines of the National Institutes of Health (NIH). In case of disagreement, interpretation differences were discussed between the authors, with a third reviewer's participation if necessary. The main characteristics evaluated were: research question, study population, uniform eligibility criteria and characteristics of the recruited population, justification of the sample size, outcome evaluation, blinding, follow-up, and statistical analysis. After the analysis, the risk of bias of each study was classified as good, fair, or poor. It is essential to mention that the topic analyzed and the number of questions used during the quality evaluation could change according to the methodology of each article (**Table 4**).

## Results

**Figure 3:**
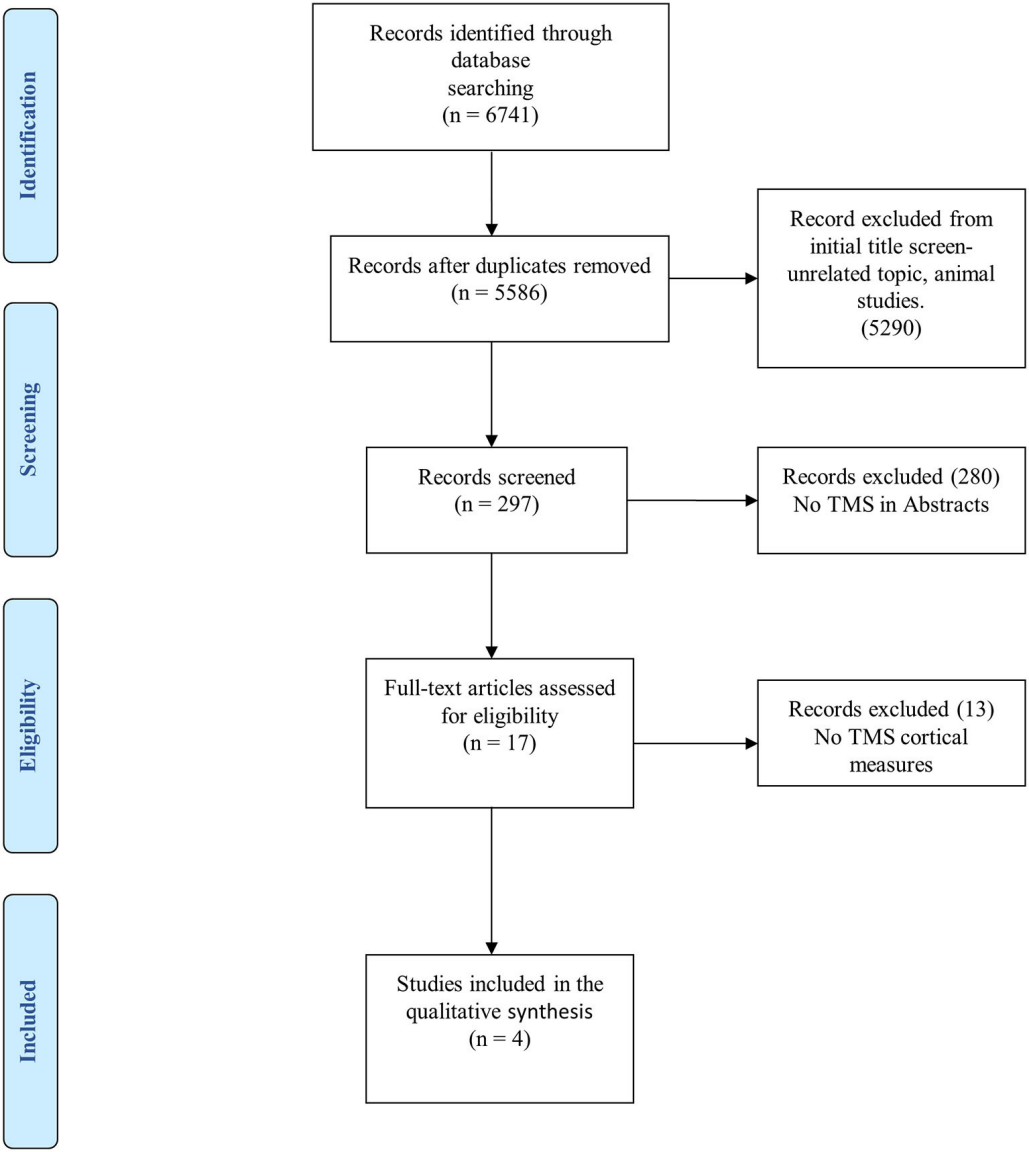
PRISMA flowchart for systematic review and meta-analysis.

The search strategy is summarized in Figure 3, as indicated in the PRISMA flowchart (129). Initially, a total of 6,741 articles were identified. After discarding animal studies and those without a title associated with the research question, this number decreased to 297. Following this, we reviewed the abstract of each study and excluded those that did not use TMS as a tool for diagnosis. Finally, after analyzing 17 articles, only four quantitatively reported CE indexed by TMS assessment.

### Demographic Characteristics of the Patients Included

The demographic and clinical characteristics of the study samples are presented in Table 2. The four reviewed articles included a total of 67 participants diagnosed with CPSP and 22 participants as controls. All subjects were adults, with a mean age of 59.5 years (SD = 6.5) in those with central pain and 55.7 years (SD = 4.5) in control subjects. CPSP was most prevalent in males, with a total of 46 participants (68.6%). The following data were extracted regarding the anatomical location of the lesion and the compromised body segment: 39 participants had hemorrhagic and 28 had ischemic lesions. Subcortical structure impairment was the most prevalent, with a total of 54 cases (31 in the thalamus, 11 in the putamen, 1 in the internal capsule, and 11 unspecified). The second most affected site was the brainstem, affected in nine subjects (three in the lateral bulb, two in the pons, and four in unspecified areas). Cortex impairment occurred in five non-reported regions. Pain level was determined using a numerical pain scale, with values between 0 and 100 in two studies and between 0 and 10 in the other two studies. The median (and interquartile range, IQ 25–75) of pain duration in months was equal to 38 (IQ = 9–47). Pain was located in the hemi-body and the upper limb in 58 cases and in the lower limb in nine subjects.

**Table 2:** Sample characteristics and central nervous system drugs used currently (*n* = 67).

**Reference**	**Study design**	**No. of patients (E/C)**	**Sex: E/C**	**Age: E/C**	**Stroke etiology**	**Stroke areas**	**Pain duration (months)**	**Baseline pain score**	**Current medication (*N*)**	**Washout period**
Hosomi et al. (126)	Before and after study	29 (21/8)	12/8 M	60 (9)/53 (10)	Hemorrhagic (17) Ischemic (4)	Thalamus (8) Putamen (7) Brain stem (4) Subcortex (2)	47 (55)	78	TCA (9), SSRI (4), BZD (6), NSAID (4), CZP (5), GBP (11), PB (1), MEX (2), ZNS (1), PHT (1)	NI
Tang et al. (125)	Cross-sectional study	28 (14/14)	12/12 M	58 (8.9)/59 (9.1)	Hemorrhagic (6) Ischemic (8)	Thalamus (8) Putamen (1) Pons (2) Lateral medulla (2) Internal capsule (1)	40 (36)	5.0 (1.4)	DXT (5), IMI (4), CZP (3), PNG (1), OXC (1)	3 days
Hasan et al. (127)	Before and after study	14 (14/0)	10 M	57	Hemorrhagic (3) Ischemic (11)	Cortex (5) Subcortex (9) Lateral medulla (1)	NI	≥4	PNG (8), CTP (7), AMP (3), TMD (1), MLT (1), NTP (1) GBP (1), KTM (1), MPN (1)	NI
Kobayashi et al. (128)	Before and after study	18 (18/0)	12 M	63 (9.9)	Hemorrhagic (13) Ischemic (5)	Thalamus (15) Putamen (3)	9 (6.8)	>70	SSRI (3), AMP (8), CBZ (2), CZP (1), GBP (1), PGN (1)	NI

*SSRI, selective serotonin reuptake inhibitor; AMP, amitriptyline; CBZ, carbamazepine; CZP, clonazepam; GBP, gabapentin; PNG, pregabalin; TCA, tricyclic antidepressant; BZD, benzodiazepine; NSAID, non-steroidal anti-inflammatory; PB, phenobarbital; MEX, mexiletine; ZNS, zonisamide; PHT, phenytoin; DXT, duloxetine; IMI, imipramine; OXC, oxcarbazepine; CTP, citalopram; TMD, tramadol; MLT, melatonin; NTP, nortriptyline; KTM, ketamine; MPN, morphine; NI, no information; M, male; E, experimental group;C, control group*.

### MEP and MT

The principal basal quantitative measures are presented in Table 3. The reviewed studies performed CE measurements in the M1 region contralateral to the compromised body segment. Two studies evaluated MEP in the first dorsal interosseous and short abductor thumb muscles (125, 126). In the other studies, the recording was performed in the anterior tibial and the first dorsal interosseous (127, 128). The cumulative mean of the RMT in the affected hemisphere evaluated in three studies was equal to 55.2% (SD = 8.9). One of these studies determined the AMT, with a mean of 58.9% (SD = 12) (128). They compared the RMT values between the unaffected hemisphere and healthy controls. The mean RMT values in different studies for the unaffected hemisphere and healthy subjects were 48.9% (SD = 10.7) and 52.8% (SD = 8.5), respectively. In the study by Hosomi et al. (126), the RMT was 65.5% (SD = 14) in the affected hemisphere compared with a threshold of 56.7% (SD = 6.5) in healthy subjects, which was a statistically significant difference (*P* = 0.035). In contrast, the other studies found no statistically significant difference when comparing the RMT of stroke patients with controls. In the studies that showed dispersion of RMT values using standard error variation, the standard deviation was calculated to quantify the median value between studies.

**Table 3:** Cortical excitability measurements.

**Reference**	**Reference muscle**	**Coil type**	**RMT**	**AMT**	**MEP amplitude**	**CSP**	**Paired pulse stimulation intensity (MT, %) and interstimulus interval**	**ICF**		**SICI**	
Hosomi et al. (126)	APB	Figure 8 coil, 70 mm	Patients: 65.5% (3)^*^	NA	Patients: 655 μV (80)	Patients: 167.9 ms (10.4)	CS: 80% RMT TS: 120% RMT 2, 4, 10, and 15 ms	Patients: 158.6% (16.5) ISIs: 10 and 15 ms	↑	Patients: 32.0% (8.7) ISIs: 2 and 4 ms	**↑**
			Controls: 56.7% (2.3)^*^		Controls: 707 μV (105)	Controls: 148.4 ms (8.7)		Controls: 168% (18.8) ISIs: 10 and 15 ms	↑	Controls: 47.3% (7) ISIs: 2 and 4 ms	**↑**
Tang et al. (125)	FDI	Figure 8 coil, 40 mm	Stroke hemisphere: 49% (12.6)	NA	NA	NA	CS: 70% RMT TS: 125% RMT 3, 5, 7, 10, 15, and 20 ms	Stroke hemisphere ISIs: 5, 7, 10, and 15 ms^*^	↑	Stroke hemisphere SICI not detected	
			Unaffected hemisphere: 49.5% (8.5)					Unaffected hemisphere ISI: 15 ms^*^	↑	Unaffected hemisphere ISI: 3 ms^*^	**↑**
			Control stroke hemisphere: 48.9% (10.3)					Control Stroke hemisphere ISIs: 10, 15, and 20 ms^*^	↑	Control Stroke hemisphere ISI: 3 ms^*^	**↑**
			Control Unaffected hemisphere: 51.6% (10.7)					Control Unaffected hemisphere ISIs: 10, 15, and 20 ms^*^	↑	Control Unaffected hemisphere ISI: 3 ms^*^	**↑**
Hasan et al. (127)	FDI ATM	Figure 8 coil, 90 mm	Stroke hemisphere: 51.1% (4.0)	NA	Stroke hemisphere: 0.99 mV (0.2)	NA	NA	NA		NA	
			Unaffected hemisphere: 48.3% (3.5)		Unaffected hemisphere: 1.12 mV (0.2)						
Kobayashi et al. (128)	FDI ATM	Figure 8 coil, 70 mm	NA	58.9% (12)	NA	NA	NA	NA		NA	

*↑ Indicates an increase or a decrease in SICI and ICF and their intensity*.

*APB, abductor pollicis brevis; FDI, first dorsal interosseous; ATM, anterior tibial muscle; RMT, resting motor threshold; MEP, motor evoked potential; AMT, active motor threshold; CSP, cortical silent period; ISI, interstimulus interval; NA, not assessed; CS, conditioning stimulation; TS, test stimulation; ICF, intracortical facilitation; SICI, short-interval intracortical inhibition*.

* *P < 0.05*.

Another basal measurement evaluated in Hasan et al. (127) and Hosomi et al. (126) was the MEP amplitude with 120% of MT. They compared the MEP measures of the injured hemisphere with the contralateral hemisphere and healthy controls. Only one study recorded MEP latency in the hemispheres of CPSP patients (127).

### Paired Pulse Measures and CSP

Tang et al. (125) noted that the variations in ICF and SICI measurements were analyzed compared to patients with CPSP and healthy individuals. Different ISIs were implemented, and the mean amplitude of the MEP of each one was normalized to the mean amplitude of the individual test stimulus. In the unaffected hemisphere, MEP was inhibited in the ISI of 3 ms (*P* = 0.01) and was facilitated in the ISI of 15 ms (*P* = 0.05). In the affected hemisphere, there was no presence of SICI, but rather facilitation in ISIs of 5, 7, 10, and 15 ms (*P* = 0.05, 0.01, 0.05, and 0.01, respectively). Similarly, in the hemispheres of healthy subjects, inhibition of MEP was demonstrated in the interval of 3 ms (*P* = 0.01) and its facilitation in 10, 15, and 20 ms (*P* = 0.01, 0.01, and 0.01 and *P* = 0.01, 0.01, and 0.05 in the control-matched stroke and unaffected hemispheres, respectively).

The CSP, SICI, and ICF were reported identically in the study of Hosomi et al. (126), being quantified among participants with central pain, comparing them with healthy individuals. Although, none of these measures showed statistically significant differences between groups, it is important to mention the trend in CSP enlargement, and the decreases in ICF and SICI in the affected hemisphere, when compared with healthy controls. Only Tang et al. (125) recorded the SAI and long-latency afferent inhibition in the hemispheres of CPSP patients.

## Bias Risk Assessment

The risk of bias, evaluated by the NIH criteria, of the four studies included in this review is presented in Table 4. In general, the main limitations of the studies are related to a lack of a clear description of the population studied, sample size calculation, and the blinding of participants or evaluators. The studies of Hasan et al. (127) and Kobayashi et al. (128) showed low methodological quality due to selection and information bias. They did not clearly describe the inclusion and exclusion criteria and the parameters for estimating the sample size and did not apply blinding to the evaluators. Although, the other two articles (125, 126) did not report a form of blinding or the parameters used to calculate the sample size, they clearly described the inclusion and exclusion criteria and, thus, were classified as having fair and good methodological quality, respectively.

**Table 4:** Assessment of risk of bias of the reviewed studies (*n* = 4).

**Methodology**	**Questions**														**Rating**
	**1**	**2**	**3**	**4**	**5**	**6**	**7**	**8**	**9**	**10**	**11**	**12**	**13**	**14**	
Cross-sectional study	Research question	The study population clearly specified and defined	The study population rate of eligibility at least 50%	Groups recruited and uniform eligibility criteria	Sample size justification	Exposure assessed prior to outcome measurement	Sufficient time frame to see an effect	Different levels of the exposure of interest	Exposure measures and assessment	Repeated exposure assessment	Outcome measures	Blinding of outcome assessors	Follow-up rate	Statistical analyses	
Tang et al. (125)	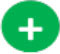	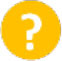	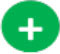	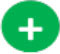	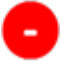	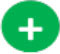	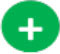	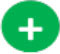	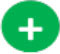	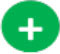	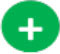	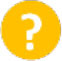	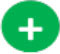	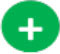	Good
Before and after study	Research question	Eligibility criteria and study population	The study participants representative of clinical populations of interest	All eligible participants enrolled	Sample size justification	Intervention clearly described	Outcome measures clearly described, valid, and reliable	Blinding of outcome assessors	Follow-up rate	Statistical analyses	Multiple outcome measures	Group-level interventions and individual-level outcome efforts	No question	No question	
Hosomi et al. (126)	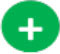	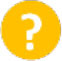	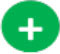	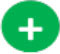	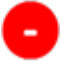	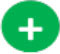	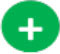	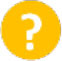	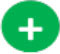	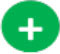	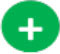	Not applicable	**–**	**–**	Fair
Hasan et al. (127)	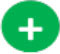	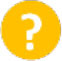	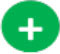	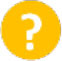	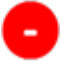	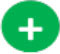	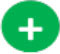	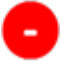	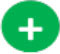	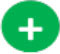	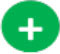	Not applicable	**–**	**–**	Poor
Kobayashi et al. (128)	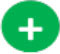	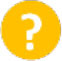	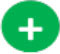	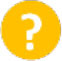	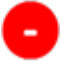	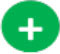	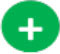	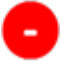	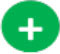	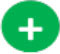	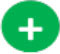	Not applicable	**–**	**–**	Poor

## Discussion

This systematic review allowed us to integrate data related to TMS measurements, somatotopic information of stroke, and clinical parameters in individuals with CPSP diagnosis. Considering the diversity of the clinical symptoms, the limited response to available treatments, and the different protocols for obtaining CE measurements, it is possible to affirm that these data are relevant to advance this field of knowledge. Because of the negative impact of CPSP on quality of life, the relevance of this subject is unquestionable, especially given the high prevalence of cerebrovascular disease in the population and the emergent demand to advance rehabilitation in stroke patients. Among the several methodological aspects, we highlighted those related to the study outcomes regarding the functional diagnosis by measures of CE. The main limitations were the heterogeneity in the parameters measuring CE, a lack of knowledge regarding CE measures as potential prognostic markers, differences in the severity and time of disease, a lack of controls with stroke and without pain, and the absence of consistent and validated diagnosis criteria. Unfortunately, it is not possible to assess the impact of drugs on CE (e.g., antidepressants and anticonvulsants), and it was not adequately reported how these CE measures relate to functional disability determined by post-stroke sequela. Finally, due to the reduced number of studies and small sample sizes, which increase the selection bias, readers are cautioned on the generalizability of the current findings.

### Integration of the Findings

The findings of this review suggest that CE measures acquired by TMS permitted us to evaluate the neurobiological systems involved in CPSP. Although, we found few studies, our analysis indicates differing results among them. In most of the assessed studies, we observed slight variabilities in the RMT and MEP. Only the study by Hosomi et al. (126) observed statistically significant differences in RMT (*P* = 0.035) and found a tendency of reduced ICF, SICI, and CSP prolongation when comparing measures of the affected hemisphere with control subjects. The decreases in ICF and SICI found in the study of Hosomi et al. (126) are likely more critical in their contrast with the findings of the study of Tang et al. (125). In assessing CE, several factors might explain intra-subject variability, including age (130, 131), sex (130), sleep deprivation (132), the severity of the disease (133), CNS drugs (i.e., anticonvulsants, antidepressants, benzodiazepines, and opioids) (134), and structural damage (65, 112). Hence, we should interpret the nature of the small variability in MT and MEP with parsimony since several reasons may explain these findings, as shown below.

One possible factor that could explain this slight difference in MT and MEP would be the severity of motor sequela, which varied from mild to moderate, which might influence the CE parameters by TMS compared to controls. This hypothesis is plausible because none of the four studies quantitatively sorted motor impairments. The studies of Tang et al. (125), Hosomi et al. (126), and Hasan et al. (127) mentioned that most of the patients had mild to moderate motor impairments or they showed rapid recovery after clinical discharge, during outpatient follow-up. Nonetheless, this is descriptive and does not permit an estimate of the severity of motor disability. Another factor influencing CE parameters is the time elapsed between the stroke and measurement acquisition. This information was found only in the study by Kobayashi et al. (128), which reported an average time of 19.7 months (SD = 9) after stroke. Moreover, Hasan et al. (127) excluded individuals whose motor response was not identified during MT measurements and the MEP amplitude from the statistical analysis; thus, this could underestimate the real mean values of the cortical measures.

Conversely, the differences in the ICF and SICI between the studies of Hosomi et al. (126) and Tang et al. (125) can be explained in other ways. Although, there are no clear reasons for these mixed findings, these divergences may be associated with the clinical differences in patients and the methodological aspects of the studies. For example, in the study by Hosomi et al. (126), the paired stimulus intensity was defined from 80% of MT for the conditioning stimulus and 120% for the test stimulus. The ISI was 2–4 ms in SICI and 10 and 15 ms in ICF. In contrast, Tang et al. (125) established intervals of 3, 5, 7, 10, 15, and 20 ms in the assessment of the ICI and facilitation. They used a conditioning stimulus at 70% of MT and 125% of this threshold for the test stimulus. Even though, it is not possible to quantify these differences in CPSP patients, according to evidence from studies in healthy subjects, these measurement intervals and thresholds for paired stimulus testing may influence the results. This hypothesis is supported by the findings of Du et al. (135), who demonstrated the existence of a range of reliability in inhibition and facilitation applying ISIs of 1–500 ms and limited to ISIs of 1–3 ms and 12–21 ms, respectively. However, the authors highlighted a high variability in the MEP amplitude among healthy individuals in the ISI windows in which facilitation and inhibition occurred. This variability may be associated with an individual response of unclear etiology, the effect of CNS drugs, or imprecision in the signal capture between stimuli. Furthermore, it has been shown that, in healthy subjects, paired stimulation protocol influenced the SICI value according to the intensity of the test stimulus (100–150% to RMT) in different situations of muscle excitability (rest and isometric abduction at the evaluated and contralateral index finger). They concluded that, regardless of the state of muscle excitability, an intensity of ~120% of RMT generated the greatest inhibition (136). Indeed, the intensities of the conditioned pulse could be associated with variations in the SICI and ICF measurements. SICI was more significant at a 3-ms ISI when a conditioning stimulus equivalent to 80% intensity of RMT was applied, whereas, ICF was more evident in ISIs of 7 and 13 ms after the application of an equivalent conditioning stimulus of 90% of the RMT (137).

Likewise, it is not possible to reject whether the disagreements in the TMS measurements present in the studies of Hosomi et al. (126) and Tang et al. (125) are related to the effect of CNS drugs. Firstly, it is important to mention that, among the four studies included in this review, only Tang et al. (125) interrupted CNS drugs 3 days before TMS evaluations. Specifically, they performed a washout period of 3 days for medications indicated for the treatment of neuropathic pain. However, the CE measures obtained must be interpreted with parsimony, considering that they were done in a neuroadaptive phase (51). For instance, different studies have reported that chronic antidepressant treatment can stimulate the upregulation of genes associated with BDNF (138) and induce BDNF expression (139), important in the process of long-term potentiation and synaptic reorganization (140). Additionally, after ~2 weeks of antidepressant treatment (141), an increase in hippocampal neurogenesis associated with their behavioral effects was observed (142), which depended on BDNF activity (143). Conversely, in the study by Hosomi et al. (126), the participants were continuously using the following medications: gabapentin (in 11 cases), tricyclic antidepressants (in 9 patients), benzodiazepines (in 6), carbamazepine (in 5), selective serotonin reuptake inhibitors (in 4), non-steroidal anti-inflammatory drugs (in 4), mexiletine (in 2), pregabalin (in 1), zonisamide (in 1), and phenytoin (in 1 patient). Although, it was not possible to find information concerning the effects of CNS drugs on the different TMS measurements of patients with CPSP, the results of studies in healthy subjects show that several of these drugs are responsible for variations in cortical activity. A review by Ziemann et al. (134) found that sodium channel blockers, such as carbamazepine and phenytoin, increase MT. These drugs produce neuronal hyperpolarization with modulation of the cortico-cortical and corticospinal axons. The carbamazepine effect on MT was reaffirmed by Darmani et al. (144) after administration of a single oral dose. Similarly, medications such as gabapentin and benzodiazepines, used in a significant number of patients in the study of Hosomi et al. (126), have been shown to affect CSP prolongation (145, 146) and ICF decrease (147, 148). According to pharmacodynamic principles, this effect may be due to GABA synthesis and the positive modulation of GABA A receptors (134, 147, 149). Like gabapentin and benzodiazepines, sertraline (a serotonin reuptake inhibitor) decreased ICF after a single oral dose of 100 mg. This effect was linked to the activation of 5-hydroxytryptamine 3 receptors (5-HT3Rs) at the cortical level, found in inhibitory interneurons, and related to indirect pyramidal cell inhibition (150, 151).

Although, the studies did not use a scale or score to stratify the degree of motor impairment, it is plausible that the severity of the motor “deficit” and sensorial alterations were the determining factors in the cortical adaptive mechanisms present in the facilitation and inhibition measurements. In the study by Hosomi et al. (126), subjects presented with mild to moderate motor impairments. Hence, the study found neurophysiological variations characteristic of chronic stroke (i.e., differences in the RMT between hemispheres). However, in the study by Tang et al. (125), patients presented with persistent sensory alterations and, in most cases, without motor impairment or with posterior motor recovery at follow-up. Additionally, they showed changes in cortical activity characterized by a decrease in intracortical inhibition. The authors defined this cortical phenomenon as being characteristic of central pain and possibly related to a dysfunction of the medial lemniscus pathway. Similar results have already been reported by Liepert et al. (152) in patients with thalamic lesions and hemihypesthesia. They did not find clinical or electrophysiological evidence of compromise in the central or peripheral motor system; however, in the affected hemisphere, they found a decrease in intracortical inhibition and an enhancement of intracortical facilitation. The authors postulated that this cortical behavior can be explained by sensory input inhibition or an excitation-limiting effect over the motor cortex.

In summary, cortical plasticity mechanisms may be related to the development and recovery of sensory–motor changes after central system injury. According to preclinical models of spinal cord injury, motor “deficits” were related to adaptive cortical changes consequential to the imbalance between the GABAergic inhibition mechanism (153) and cortical excitatory stimulus release (154). These changes likely are necessary to remap the injured brain region and could be associated with the onset of neuropathic pain (155). This excitatory response influences the expression of Nav 1.3-dependent sodium-voltage channels associated with increased sensitivity to stimuli and central pain development (156). Hence, the importance of the cortical reorganization process in pain pathophysiology has already been confirmed. A direct association between the degree of reorganization of the primary somatosensory cortex and the pain intensity in individuals with spinal cord injury and neuropathic pain has been reported (157). However, patients with spinal cord injury showed an analgesic response to different therapies (e.g., virtual walking techniques) independently of the spinal cord injury level (158). Therefore, these effects were associated with changes in somatosensory cortex organization (159). From this set of data, it is possible to suppose that there are sensory and motor manifestations consequent to the same structural lesions in the CNS, which concurs with specific adaptive cortical changes, but with different evolution and responses to therapeutic approaches due to a neuroplasticity state influenced by varying degrees of physiological, biological, and social factors (131, 160, 161).

## Future Directions

Specialized centers allied to therapeutic advancements, such as thrombolysis and thrombectomy, led to scientific advances in the acute clinical treatment of stroke (162). Although, this advancement is of inestimable relevance, considering that stroke is the second leading cause of death by chronic disease in the world (1), it is vitally important that our efforts turn to seeking biomarkers that can assist in diagnosis, prognosis, and response predictors in the course of rehabilitation. In this scenario, CE measurements can help in understanding neuroplasticity processes, fundamental in the neurorehabilitation of somatosensorial systems. Although, pharmacological treatment of chronic neuropathic pain has grown substantially, together with the criteria to define neuropathic pain conditions (163, 164), evidence-based treatments of CPSP remain scarce. Thus, central pain clinical management is done empirically, by indication of the specialist. The lack of adequate parameters to evaluate clinical response and the lack of neurofunctional parameters to assess the therapeutic course make it difficult to open new therapeutic avenues. Thus, this review provides data pointing out ways that may help in the evaluation and research planning to seek rational treatment. The current CPSP classification is based mainly on descriptors, signs, and areas of the body where pain symptoms are referred to topographically, combined with information on anatomical impairment (e.g., neuroimaging data, such as functional magnetic resonance imaging). Few studies have evaluated CPSP with a perspective that integrates neurophysiological parameters with clinical, somatosensory, cognitive, and emotional symptoms. It is assumed that substantial improvements in the treatment of chronic pain after stroke may come from coordinated strategies that can identify specific mechanisms, aligning them with a biopsychosocial approach according to the ACTTION-American Pain Society Taxonomy (AAPT), which includes the following dimensions: (1) essential diagnostic criteria (e.g., symptoms, signs, diagnostic tests, and chronic pain condition); (2) standard features (e.g., location, temporal qualities, descriptors, fatigue, and numbness); (3) medical comorbidities (e.g., major depression); (4) neurobiological, psychosocial, and functional consequences; and (5) neurobiological and psychological mechanisms, as well as risk and protective factors (i.e., central sensitization, descending pain inhibitory system dysfunction, and somatosensory amplification) (165). In this context, more studies are needed to better understand an integrative view of the interactions among the different variables of the clinical picture, including the injury location, the degree of motor impairment, and the duration of CPSP.

## Limitations

We know that this review has important limitations due to the limited number of articles available and the critical heterogeneity between them, preventing definitive conclusions about TMS parameters as markers of the neuroplasticity involved in CPSP. Other important factors that could hinder the interpretation of cortical measures are the heterogeneity between the protocols used, inappropriate control over the impacts of drugs, the lack of data concerning the time of disease, and the severity of clinical symptoms. It is also important to mention that the studies did not have a control condition for stroke patients without pain and present a lack of correlation analyses between pain levels and CE measures. These factors make it difficult to detangle the stroke structural effect only vs. the changes leading to pain.

Despite these limitations, one needs to consider that this is an emerging research area that may significantly impact public health, given its relevance in advancing stroke patient rehabilitation. From this review, we have identified several aspects of the risk of bias, such as the small sample size and heterogeneity, which reduce the strength of our conclusions. Thus, we need more studies to clarify CE measurement properties as diagnostic and prognostic biomarkers to predict the therapeutic response of CPSP. Although, TMS may be a useful method to answer these questions, the results are preliminary. According to this, our review points out several methodological aspects to consider in future studies, such as the establishment of a better description of the clinical symptoms related to impairments of somatosensory systems, the time elapsed between stroke and CPSP assessment, the definition of CNS lesion extent by neuroimaging methods, and current use of drugs with active effects on the CNS. It is also important to have a detailed description of the methods used to measure CE parameters. Hence, longitudinal data are important to monitor the effects of multiple confounding variables that are not easily controlled among patients, such as genetic, clinical, and environmental characteristics. Finally, we need studies to understand the potential benefit of therapies used to mitigate symptoms, including pharmacological and non-pharmacological interventions, behavioral techniques, or physical rehabilitation, in isolation or associated with magnetic stimulation.

## Conclusions

The results of this systematic review indicate a significant heterogeneity among the studies examined, which limits the establishment of definitive conclusions on CE parameters as a diagnostic measure, prognostic indicator, or surrogate biomarker related to CPSP. Despite the limited number of studies that prevented us from conducting a meta-analysis, the dataset of this systematic review provides evidence to improve our understanding of the physiopathology of CPSP. Additionally, the studies examined provide support to construct a framework for diagnosis and to improve the methodological quality of future research on somatosensory sequelae after stroke. Furthermore, they offer a way to integrate dysfunctional neuroplasticity markers assessed indirectly by neurophysiological measures with their correlated clinical symptoms.

## Data Availability Statement

The original contributions presented in the study are included in the article/supplementary material, further inquiries can be directed to the corresponding author/s.

## Author Contributions

DB, MT, and WC conceived and designed the study, participated in the data collection, performed the statistical analysis, and coordinated and drafted the manuscript. IT and FF drafted the work or revised it critically for important intellectual content. All authors have agreed and approved the final version of this work.

## Conflict of Interest

The authors declare that the research was conducted in the absence of any commercial or financial relationships that could be construed as a potential conflict of interest.

## Publisher's Note

All claims expressed in this article are solely those of the authors and do not necessarily represent those of their affiliated organizations, or those of the publisher, the editors and the reviewers. Any product that may be evaluated in this article, or claim that may be made by its manufacturer, is not guaranteed or endorsed by the publisher.
